# Effect of BLUP prediction on genomic selection: practical considerations to achieve greater accuracy in genomic selection

**DOI:** 10.1186/1753-6561-5-S7-P49

**Published:** 2011-09-13

**Authors:** Patricio Munoz, Marcio Resende, Gary Peter, Dudley Huber, Matias Kirst, Tania Quesada

**Affiliations:** 1 Plant Molecular and Cellular Biology Program, University of Florida, Gainesville, FL, USA; 2 Genetic and Genomics Graduate Program, University of Florida, Gainesville, FL, USA; 3 School of Forestry Resources and Conservation, Genetic and Genomics Graduate Program, Plant Molecular and Cellular Biology Program, University of Florida, Gainesville, FL, USA; 4 School of Forestry Resources and Conservation, University of Florida, Gainesville, FL, USA

## Background

Prediction of breeding values (BV) using only genotypic information is the final goal of Genomic Selection (GS) [1]. Commonly, BV prediction from traditional BLUP analysis is the input for constructing GS prediction models, and GS predicted BVs are correlated with traditional BLUP BVs to estimate the accuracy of GS models. The use of GS in plant breeding depends on the accuracy of the GS models to predict the BVs. Therefore, better accuracy and less bias in traditional BLUP BVs should improve the final accuracy of GS predictions. Such improvements in GS predictions are not due to GS modeling itself, but rather to the reduced noise in the BLUP BV used as input.

Improvements in BLUP BV can be obtained simply by correcting errors in the pedigree [2] or using more complex approaches, such as applying a realized relationship matrix (RRM) in the BLUP prediction as an alternative to the relationship matrix (A) based on expected values derived from the pedigree [3]. Misspecification of effects in BLUP models tends to produce upward bias in the BV estimates, which also impact GS accuracy [4]. In addition, not correcting with the additive-genetic relationship information in the GS prediction model leads to overestimates in accuracies due to inadequate accounting for confounding genetic relationships found in the training population [5]. The inflated accuracy cannot be exploited in future generations and should be guarded against.

Our objective was to use real data to study the effect on the GS accuracy from 1) pedigree errors, 2) incorporation of the RRM in the BLUP analysis, 3) misspecification of non-additive effects in the BLUP analysis and 4) the effect of ignoring the additive-genetic relationship in the GS prediction model.

## Methods

Height (HT) was measured in one field test containing 860 clonally propagated loblolly pine trees (~8 ramets per genotype) derived from 32 parents crossed in a circular mating design. The population was genotyped using the Illumina Infinium™ assay (Illumina, San Diego, CA) with 7,216 SNPs. A total of 3,938 SNPs were selected for use in GS based on frequency of polymorphism across genotypes, quality and reliability of the reads. SNP markers were used to estimate the RRM following a recently published method [3] where identity by descent is determined relative to a base population. RRM values were adjusted as recommended [6] to obtain less biased variance estimations. Based on the RRM, a new pedigree was constructed.

Several BLUP models were fit in ASReml to study the following effects:

Model 1: Additive + non-additive effects model – original pedigree

Model 2: Additive + non-additive effects model – new pedigree (expected A matrix)

Model 3: Additive model – new pedigree based (expected A matrix)

Model 4: Additive model – RRM (observed A matrix)

The BVs obtained from models 1-4 were deregressed and used to construct GS prediction models with GBLUP [1]. Additionally, two GS prediction models were constructed based on the raw BVs (not deregressed) from models 3 and 4 to study the effect of ignoring the additive-genetic relationships in the training population when constructing the GS model.

## Results and discussion

**Figure 1:**
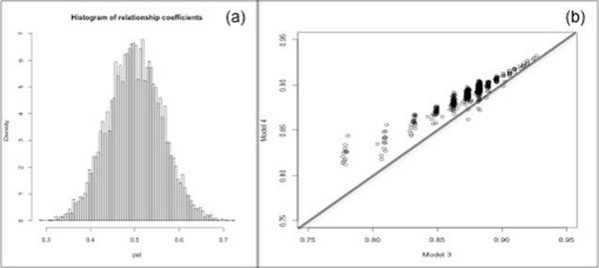
(a) Distribution of relationship values around the expected mean (0.5) for full-sib individuals n=6475, SD=0.06; (b) Accuracy of BLUP prediction in pedigree-based (Model 3) and RRM-based (Model 4) analysis

The RRM among 6475 full-sib pairs (Figure 1a) showed a normal distribution of relationship coefficients around the expected value.

As expected, when the RRM was used to correct the original pedigree [3] the accuracy of the BLUP predictions increased from 0.80 to 0.85 (Table 1), and GS accuracy improved from 0.64 to 0.77 [4]. When the RRM was used directly, instead of the corrected pedigree accuracy of the BLUP, the BVs improved (Figure 1b). Improved BLUP BV estimates also resulted in the improvement of the accuracy of GS predictions from 0.58 to 0.60. The same results were obtained when the additive model was compared with the full model, indicating that misspecification of effects in the BLUP model will cause a decrease in the GS accuracy [5]. In addition, as shown [6] ignoring the additive-genetic relationship dramatically inflates GS accuracy from 0.58 to 0.87 and from 0.60 to 0.88 for Models 3 and 4 respectively.

**Table 1:** Heritability, BLUP and GS accuracy for models 1, 2, 3 and 4.

ModelNumber	Heritability^1^	BLUP^2^Accuracy^2^	Deregressed	GS^3^Accuracy^3^
1	0.26	0.80	Yes	0.64
2	0.31	0.85	Yes	0.77
3	0.34	0.87	Yes	0.58
3	0.34	0.87	No	0.87
4	0.36	0.89	Yes	0.60
4	0.36	0.89	No	0.88

^1^ Narrow sense heritability, ^2^ Average of accuracy among all clones, ^3^ correlations between BLUP BVs and GS-BVs prediction

## Conclusions

To maximize the true accuracy of GS, it is recommended: 1) construct a RRM for the training population that should be used to correct the pedigree and to predict the BLUP BVs, 2) correct for non-additive effects if using a family related training population, and 3) deregress BVs prior to use as input for construction of GS prediction models.

## References

[B1] Meuwissen T Hayes B Goddard M Prediction of total genetic value using genome-wide dense marker maps Genetics 2001 157 1819 1829 11290733 10.1093/genetics/157.4.1819 PMC1461589

[B2] Sander K Bennewitz J Kalm E Wrong and missing sire information affects genetics gain in the angeln dairy cattle population J Dairy Sci 2006 89 315 321 10.3168/jds.S0022-0302(06)72096-3 16357295

[B3] Powell J Visscher P Goddard M Reconciling the analysis of IBD and IBS in complex trait studies Nat Rev Genet 2010 11 800 805 10.1038/nrg2865 20877324

[B4] Lee S Goddard M Visscher P van der Werf J Using the realized relationship matrix to disentangle confounding factors for the estimation of genetic variance components of complex traits Genet Sel Evol 2010 42 22 10.1186/1297-9686-42-22 20546624 PMC2903499

[B5] Habier D Fernando R Dekkers J The impact of genetic relationship information on genomic-assisted breeding values Genetics 2007 42 5 10.1534/genetics.107.081190 PMC2219482 18073436

[B6] Yang J Benyamin B McEvoy B Gordon S Henders A Nyholt D Madden P Heath A Martin N Montgomery G Goddard M Visscher P Common SNPs explain a large proportion of the heritability for human height Nat Genet 2010 42 565 571 10.1038/ng.608 20562875 PMC3232052

